# Over-expression of NFYB affects stromal cells reprogramming and predicts worse survival in gastric cancer patients

**DOI:** 10.18632/aging.204294

**Published:** 2022-09-22

**Authors:** Tailiang Lu, Chenglong Li, Cailing Xiang, Yongqiang Gong, Wei Peng, Futao Hou, Chaowu Chen

**Affiliations:** 1 Department of General Surgery, Hunan Provincial People’s Hospital, The First-Affiliated Hospital of Hunan Normal University, Changsha, Hunan Province, China

**Keywords:** NFYB, cancer-associated fibroblasts (CAFs), stromal cells reprogramming, gastric cancer, prognosis

## Abstract

Gastric cancer (GC) is the fifth most common cancer worldwide and the third most fatal. Cancer-associated fibroblasts (CAFs) play an essential role in promoting the occurrence and development of gastric cancer in all stages. NFYB is highly expressed in multiple tumors and promotes tumor invasion, metastasis, and drug resistance, but its role in the occurrence and development of gastric cancer remains unclear. Hence, we used TCGA, TIMER, Kaplan-Meier Plot, and UALCAN databases to analyze the expression of NFYB in pan-cancers and assess its clinical prognostic value. We found that high expression of NFYB may be a promising prognostic biomarker in patients with gastric cancer. High expression of NFYB was associated with high T stage, high histological grade, diffuse gastric cancer, and early-onset GC. Moreover, High expression of NFYB was associated with CAFs infiltration in the GC microenvironment. The prognosis of GC patients with high expression of NFYB and high infiltration of CAFs was worse. Therefore, NFYB may serve as a potential prognostic biomarker in patients with GC.

## INTRODUCTION

Gastric cancer (GC) is a common gastrointestinal tumor. The number of new cases of GC in China accounts for 43.9% of the global total each year, posing a severe threat to the health of the Chinese people [1]. Radical surgical resection is still the cornerstone of gastric cancer treatment. The development of minimally invasive surgery, new targeted drugs, and immunotherapy have brought breakthroughs in treating gastric cancer patients. However, the overall therapeutic effect of gastric cancer is still not satisfactory. Both the individual heterogeneity of GC patients and the low response rate to immunotherapy affected the prognosis of GC patients. It is of great clinical significance to explore the new molecular mechanism of GC and to find new therapeutic targets for GC.

The extracellular matrix and various mesenchymal cells constitute the tumor stromal microenvironment [2]. Cancer-associated fibroblasts (CAFs) are the most abundant cell type of mesenchymal cells [3, 4]. Many CAFs in tumor tissue create a suitable environment for tumor development. CAFs play an essential role in cancer: CAFs can not only suppress the function of immune cells by secreting a variety of cytokines or metabolites to promote the development, invasion, and metastasis of tumors but also shape the external tumor stromal and form the permeation barrier to prevent the deep infiltration of drugs and immune cells into tumor tissues, thus reducing the therapeutic effect of tumor [3]. Therefore, Regulating CAFs or overcoming their barrier effect to control tumors is a new approach to tumor therapy [5]. Metastasis was the leading cause of poor prognosis and low survival rate in GC patients, which is responsible for about 60% of GC deaths [5]. Many studies have shown that CAFs can directly or indirectly promote the migration and invasion of GC cells by releasing growth factors or cytokines. CAFs can induce GC invasion and metastasis through multiple cascade pathways by regulating miRNA expression [6]. Wu et al.’s study showed that CAFs could secrete a large amount of IL-6 to induce EMT by activating the JAK2/STAT3 pathway, increase the migration of gastric cancer cells, and promote the development of GC [7]. Shen et al. demonstrated that vascular cell adhesion molecule 1(VCAM1) derived from CAFs could interact with integrin αVβ1/5 in GC cells to promote tumor invasion *in vivo* and *in vitro* [8]. CAFs are expected to be a potential diagnostic marker and therapeutic target for gastric cancer. Currently, the method to treat cancers is being explored by targeting CAFs [9]. However, there are still many challenges, such as the lack of specific markers of CAFs, which makes it difficult to make specific targeting strategies, and the functional heterogeneity and polarization dynamics of CAFs. So, it is necessary to have a deeper understanding of the therapeutic response to CAFs and more accurate targeting methods.

NF-Y is a ubiquitous heterotrimer transcription factor with a binding affinity with CCAAT consensus motifs and is one of the most common cis-acting elements in promoter and enhancer regions of eukaryotic genes [10, 11]. NF-Y consists of three subunits, namely the regulatory subunits NF-YA, NF-YB, and NF-YC, all of which require binding to CCAAT [12]. More and more studies have shown that the NF-Y gene family drives the transcription of many cell cycle regulation genes and plays a crucial role in proliferation regulation [13]. NFYB plays a vital role in many biological processes, including cell proliferation, senescence, and apoptosis. Studies have shown that NFYB can enhance the activity of STK33 and promote cisplatin resistance in diffuse large B cell lymphoma [14]. NFYB induces high expression of E2F1 and promotes oxaliplatin resistance in colorectal cancer by enhancing the CHK1 signaling pathway [15]. In head and neck squamous cell carcinoma, single-cell RNA-seq deconvolution revealed that NF-YA was associated with cancer-associated fibroblasts (CAFs) and p-EMT cells (populations with metastatic potential) [16]. The role of NFYB in the occurrence and development of GC remains unclear. Therefore, we used bioinformatics methods to analyze the expression of NFYB in the GC cohort and its correlation with clinicopathology and the prognosis of GC patients. Meanwhile, we analyzed the possible mechanism of NFYB affecting the infiltration of CAFs in GC.

## RESULTS

### NFYB is highly expressed in gastric cancer tissues

**Figure 1:**
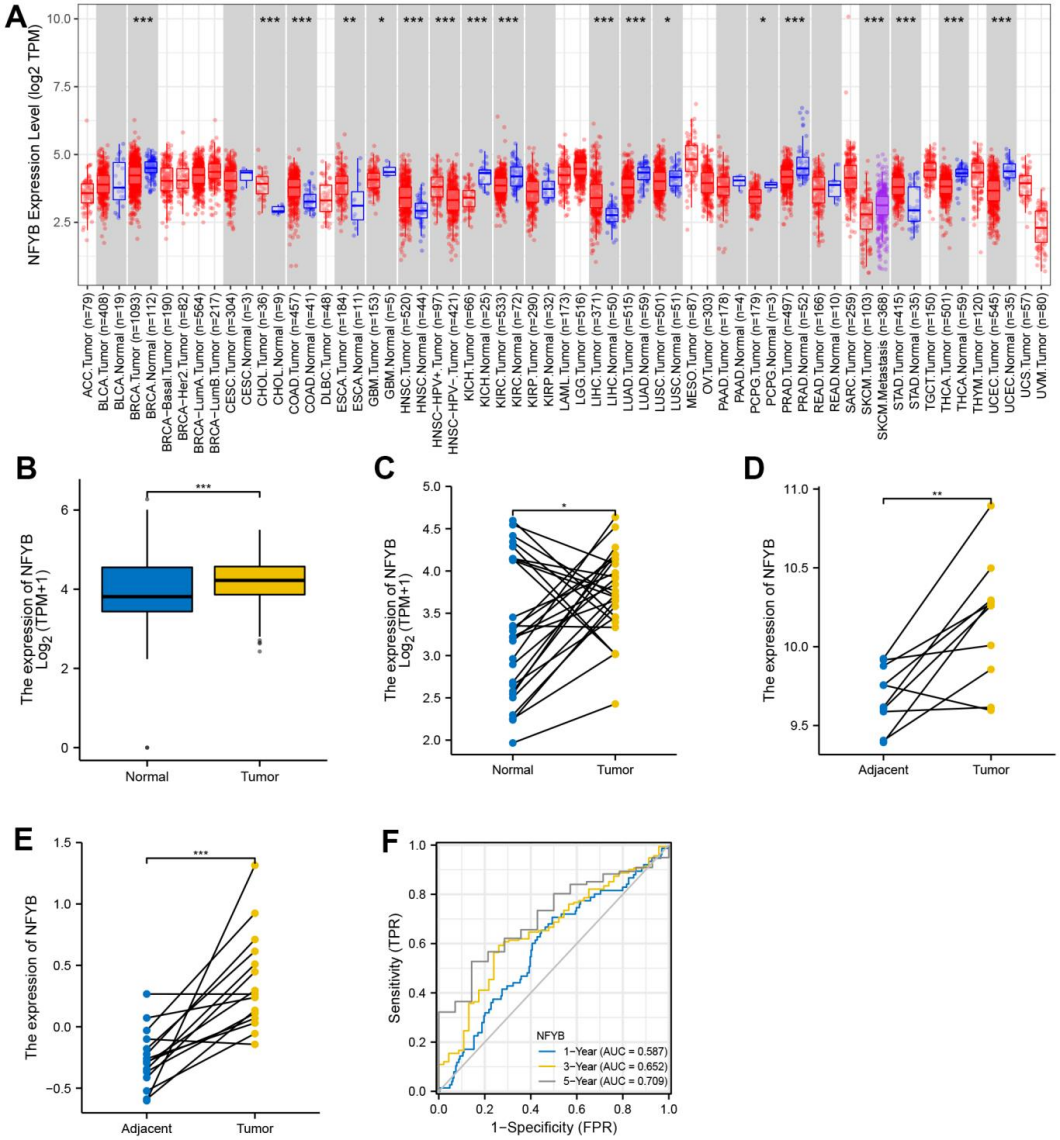
**NFYB is highly expressed in gastric cancer tissues.** (**A**) The expression of NFYB in pan-cancer of TCGA. (**B**) The expression of NFYB in non-paired GC and stomach tissue of TCGA and GETx. (**C**) The expression of NFYB in paired GC and adjacent normal tissue of TCGA. (**D**) The expression of NFYB in paired GC and adjacent normal tissue of GSE79973. (**E**) The expression of NFYB in paired GC and adjacent normal tissue of GSE118916. (**F**) The time dependence ROC curve analysis of NFYB.

We used the TIMER database to analyze TCGA’s pan-cancer RNA-SEQ expression data and explore the expression of NFYB in various tumors (Figure 1A). The results showed that NFYB expression was significantly higher in Cholangiocarcinoma (CHOL), colon cancer, esophageal cancer, head and neck cancer, hepatocellular carcinoma, and gastric cancer than in adjacent normal tissues. It was significantly lower expressed in breast cancer, glioma, kidney cancer, pheochromocytoma and paraganglioma, prostate cancer, skin melanoma, and endometrial cancer than in normal adjacent tissues. This suggests that NFYB may have different functions in different types of cancer. To further explore the differential expression of NFYB in GC, RNAseq data of the TCGA-STAD cohort and gastric tissue in the GETx database were downloaded and merged after removing the batch effect. And then, the differential expression of NFYB in GC was analyzed again using the R software. The results showed that NFYB was significantly overexpressed in gastric cancer tissues compared with normal gastric tissues (Figure 1B), which was consistent with the results of TIMER data. We further analyzed NFYB expression using paired GC and adjacent normal tissue samples from TCGA and GEO. NFYB was significantly over-expressed in GC tissues than in adjacent ones (Figure 1C–1E). The time dependence ROC curve analysis of NFYB in GC showed that the AUC of 1, 3, and 5 years were 0.587, 0.652, and 0.709, respectively. The above data suggest that NFYB was significantly overexpressed in GC tissues, which may be a potential molecular biomarker for GC diagnosis.

### Correlation between the expression of NFYB and clinicopathological features of GC

**Figure 2:**
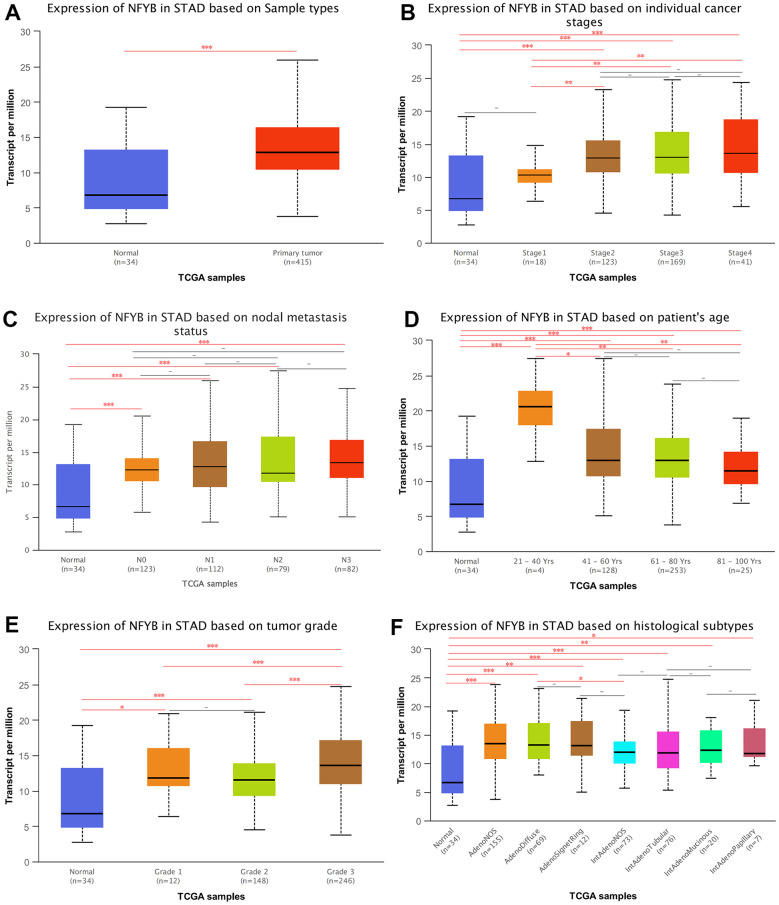
**Correlation between the expression of NFYB and clinicopathological features of GC.** (**A**) Expression of NFYB in STAD based on Sample types. (**B**) Expression of NFYB in STAD based on individual cancer stages. (**C**) Expression of NFYB in STAD based on nodal metastasis status. (**D**) Expression of NFYB in STAD based on patient’s age. (**E**) Expression of NFYB in STAD based on tumor grade. (**F**) Expression of NFYB in STAD based on histological subtypes.

We used the UALCAN database to explore the correlation between NFYB expression and clinicopathological features of GC. The total expression of NFYB in GC tissues was significantly higher than that in normal gastric tissues (Figure 2A). The expression of NFYB in stage I gastric cancer was not significantly different from that in normal tissues, but it was significantly up-regulated in stage II, III, and IV GC tissues compared with normal and stage I GC tissues (Figure 2B). These results indicated that NFYB might be related to the development, invasion, and metastasis of gastric cancer. There was no significant difference in the expression of NFYB in GC tissues of different N stages (Figure 2C). The expression of NFYB in 21-40 year old GC patients was significantly higher than that in older GC patients (Figure 2D), suggesting that NFYB may play a role in the occurrence and development of early-onset gastric cancer. By comparing the expression of NFYB in GC tissues of different histological grades, we found that NFYB expression was higher in poorly differentiated GC (Figure 2E). Meanwhile, our analysis showed that the expression of NFYB was higher in diffuse gastric cancer than in intestinal gastric cancer (Figure 2F). These results suggested that high NFYB expression may be associated with a poor prognosis of GC. As shown in Table 1, the expression of NFYB was correlated with T stage, gender, histological grade, and primary site of GC. Altogether, the NFYB was correlated with T stage, histological grade, diffuse gastric cancer, and early-onset of GC.

**Table 1:** The correlations between NFYB mRNA expression and the clinicopathological characteristics of GC patients.

**Characteristic (n)**	**Low expression of NFYB (187)**	**High expression of NFYB (188)**	***p***
T stage, n (%)			< 0.001*
T1	16 (4.4%)	3 (0.8%)	
T2	47 (12.8%)	33 (9%)	
T3	87 (23.7%)	81 (22.1%)	
T4	34 (9.3%)	66 (18%)	
N stage, n (%)			0.292
N0	60 (16.8%)	51 (14.3%)	
N1	51 (14.3%)	46 (12.9%)	
N2	39 (10.9%)	36 (10.1%)	
N3	30 (8.4%)	44 (12.3%)	
M stage, n (%)			1.000
M0	163 (45.9%)	167 (47%)	
M1	12 (3.4%)	13 (3.7%)	
Pathologic stage, n (%)			0.058
Stage I	35 (9.9%)	18 (5.1%)	
Stage II	53 (15.1%)	58 (16.5%)	
Stage III	69 (19.6%)	81 (23%)	
Stage IV	16 (4.5%)	22 (6.2%)	
Gender, n (%)			0.045*
Female	57 (15.2%)	77 (20.5%)	
Male	130 (34.7%)	111 (29.6%)	
Histologic grade, n (%)			< 0.001*
G1	5 (1.4%)	5 (1.4%)	
G2	90 (24.6%)	47 (12.8%)	
G3	87 (23.8%)	132 (36.1%)	
Anatomic neoplasm subdivision, n (%)			0.008*
Antrum/Distal	60 (16.6%)	78 (21.6%)	
Cardia/Proximal	28 (7.8%)	20 (5.5%)	
Fundus/Body	62 (17.2%)	68 (18.8%)	
Gastroesophageal Junction	30 (8.3%)	11 (3%)	
Other	3 (0.8%)	1 (0.3%)	
Age, median (IQR)	67 (58, 73)	68 (58.5, 73.5)	0.966

*P<0.05 was considered significant.

### High expression of NFYB was associated with a poor prognosis of GC

**Figure 3:**
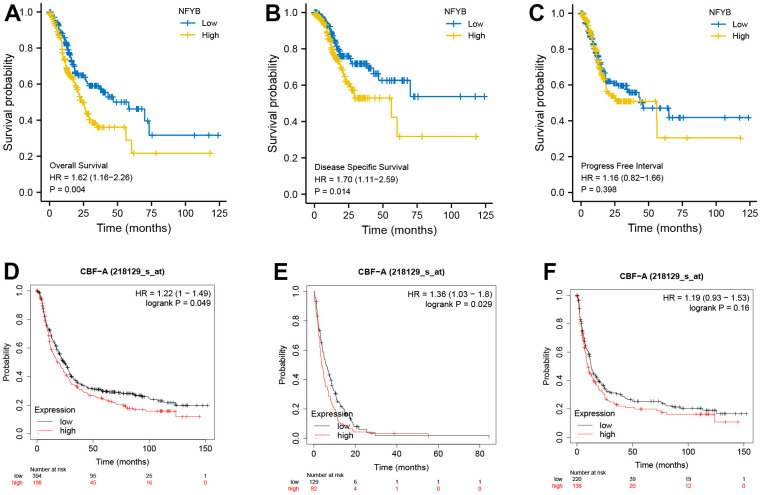
**High expression of NFYB is associated with poor prognosis of GC.** (**A**) Overall survival analysis based on NFYB expression of GC patients in TCGA data. (**B**) Disease-free survival analysis based on NFYB expression of GC patients in TCGA data. (**C**) Progression-free survival analysis based on NFYB expression of GC patients in TCGA data. (**D**) Overall survival analysis based on NFYB expression of GC patients in the Kaplan-Meier plotter database with GEO datasets. (**E**) Disease Free Survival based on NFYB expression of GC patients in the Kaplan-Meier plotter database with GEO datasets. (**F**) Progression-free survival analysis based on NFYB expression of GC patients in the Kaplan-Meier plotter database with GEO datasets.

The previous analysis found that NFYB was highly expressed in gastric cancer tissues and was associated with prognostic risk factors of gastric cancer (high T stage, poor differentiation, and diffuse gastric cancer). This suggested that the expression of NFYB may be related to the prognosis of gastric cancer patients. Hence, we downloaded the survival data of the TCGA-STAD cohort and analyzed the relationship between the expression of NFYB and the prognosis of GC patients using the “survival” R package. Meanwhile, the Kaplan-Meier Plotter database was used to verify the relationship between NFYB expression and prognosis of GC by analyzing the GEO array datasets. The results showed that high NFYB expression was associated with poor OS (Figure 3A, 3D) and DFS (Figure 3B, 3E) survival of GC patients but not correlated with PFS survival (Figure 3C, 3F). Therefore, high expression of NFYB was associated with a poor prognosis of GC.

### NFYB co-expressed genes were associated with lipid metabolism and the immune signaling pathways

**Figure 4:**
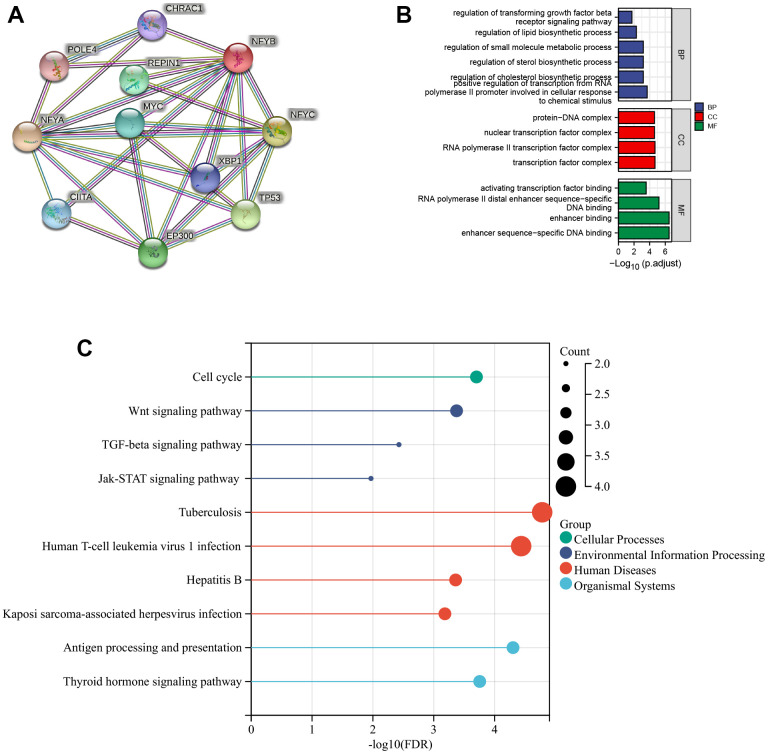
**Functional enrichment analysis of NFYB co-expressed genes.** (**A**) PPI network of NFYB co-expressed genes. (**B**) The histogram of GO Enrichment Analysis of NFYB co-expressed genes. (**C**) The bubble diagram of KEGG pathway Enrichment Analysis of NFYB co-expressed genes.

We performed protein-protein interaction network analysis using a string database to explore NFYB interacting proteins and their possible functions. The minimum interaction score was set as 0.9, and the PPI Enrichment p-value was less than 0.05. The results showed that the NFYB protein interaction network contained 11 genes with 23 edges, and the PPI enrichment p-value was 0.0033 (Figure 4A). These genes include NFYA POLE4, NFYB, EP300, NFYC, TP53, MYC, XBP1, REPIN1, CHRAC1, and CIITA. We performed the GO and KEGG pathways enrichment analysis of NFYB interacting proteins. The results showed these genes are closely related to gene transcription regulation and lipid metabolism. It is also closely related to the Wnt signaling pathway, TGFβ signaling pathway, and JAK-Stat signaling pathway (Figure 4B, 4C). Integrally, NFYB co-expressed genes were shown to be closely related to lipid metabolism as well as the immune signaling pathways.

### NFYB was associated with gastric cancer stromal cell reprogramming

**Figure 5:**
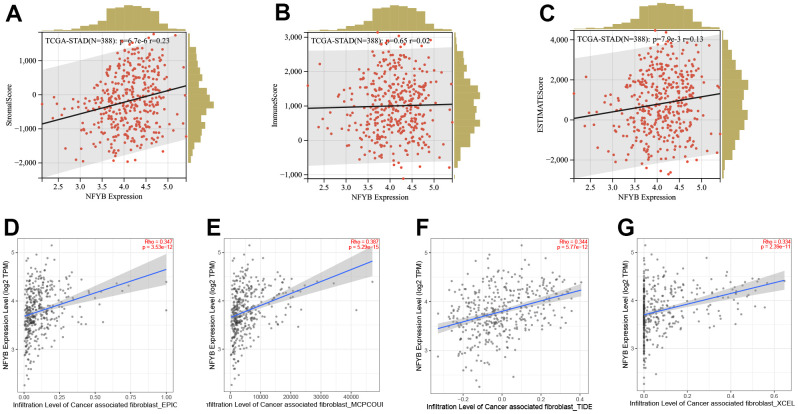
**NFYB is associated with gastric cancer stromal cell reprogramming.** (**A**) The correlation between the expression of NFYB and the stromal score of GC. (**B**) The correlation between the expression of NFYB and the immune score of GC. (**C**) The correlation between the expression of NFYB and ESTIMATE score of GC. (**D**) The correlation between the expression of NFYB and CAFs infiltration of GC evaluated by EPIC. (**E**) The correlation between the expression of NFYB and CAFs infiltration of GC evaluated by MCO-counter. (**F**) The correlation between the expression of NFYB and CAFs infiltration of GC evaluated by TIDE. (**G**) The correlation between the expression of NFYB and CAFs infiltration of GC evaluated by xCELL.

We used the ESTIMATE algorithm to evaluate the immune, stromal, and ESTIMATE scores of the TCGA-STAD cohort and analyzed the correlation between NFYB expression and those scores. Results NFYB expression was positively correlated with GC stromal score but not with the immune score or ESTIMATE Score (Figure 5A–5C). This suggested that NFYB may reprogram stromal cells in GC patients. Therefore, we further analyzed the correlation between the expression of NFYB and infiltration of CAFs through the TIMER2.0 database. The results showed that the infiltration degree of CAFs in GC, which was evaluated by xCELL, McP-counter, EPIC, and TIDE algorithm, respectively, were all positively correlated with the expression of NFYB (Figure 5D–5F). Hence, NFYB was associated with gastric cancer stromal cell reprogramming.

### NFYB was correlated with the CAFs biomarkers

**Figure 6:**
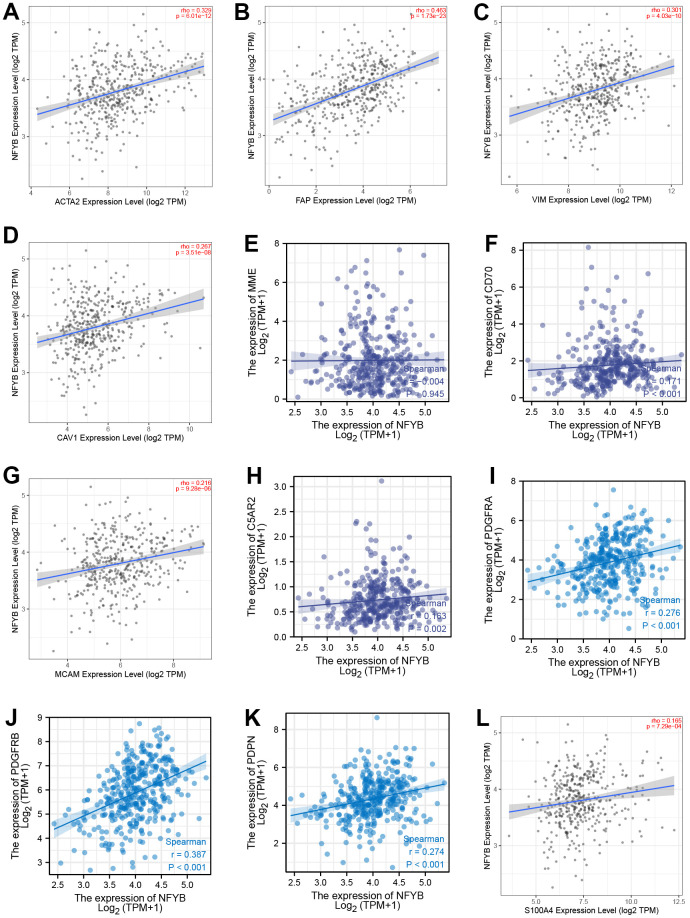
**Correlation between the expression of NFYB and CAFs biomarkers.** The correlation between the expression of NFYB and α-SMA (**A**), FAP (**B**), VIMENTIN (**C**), CAV1 (**D**), CD10 (**E**), CD70 (**F**), CD146 (**G**), GPR77 (**H**), PDGFRA (**I**), PDGFRB (**J**), PDPN (**K**), S100A4 (**L**).

To further explore the relationship between NFYB and CAFs, we analyzed the correlation between the expression of NFYB and the CAFs’ molecular markers. Those markers are listed in Table 2. The expression of NFYB was positively correlated with most CAFs molecular markers (Figure 6A–6L and Table 2). The expression of FAP, α-SMA, and Vimentin was more strongly correlated with NFYB (Figure 6A–6C and Table 2), and these markers were closely related to tumor invasion and metastasis.

**Table 2:** The correlations between NFYB mRNA expression and the CAFs markers.

	**Markers**	**Correlation**	***p-value***
Neutral biomarkers with dual functions	α-SMA	0.329	***
	S100A4	0.165	***
pro-tumorigenesis biomarkers	FAP	0.463	***
	PDGFRα/β	0.276/0.387	***
	PDPN	0.274	***
	CD70	0.171	***
	Vimentin	0.301	***
	GPR77	0.163	**
	CD10	0.004	0.943
	CD74	0.101	0.05
tumor-suppressive biomarkers	CD146	0.216	***
	CAV1	0.267	***
	SAA3P	0.016	0.747

***means *P*<0.001;**means *p*<0.01,*means *p*<0.05.

### High expression of NFYB and high infiltration of CAFs predicted a worse prognosis for GC patients

**Figure 7:**
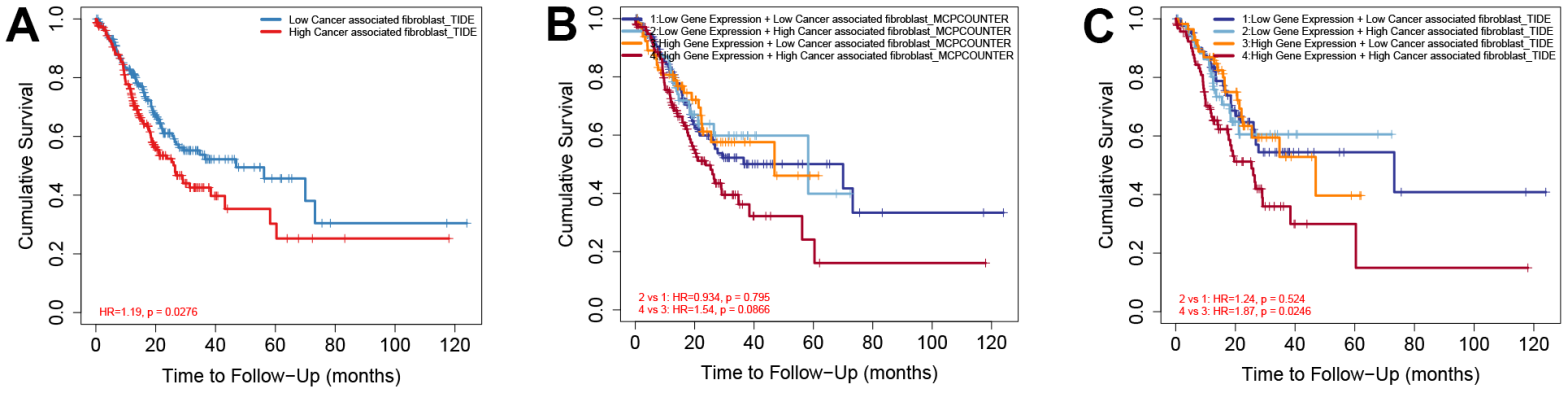
**High expression of NFYB and high CAF infiltration of CAFs predicted a worse prognosis for GC patients.** (**A**) Overall survival analysis based on CAFs infiltration in GC patients. (**B**) Overall survival analysis based on NFYB expression and CAFs infiltration calculated by MCP-counter in GC patients. (**C**) Overall survival analysis based on NFYB expression and CAFs infiltration calculated by TIDE in GC patients.

We further explored the relationship between the CAFs infiltration and the prognosis of GC patients, as well as the effect of NFYB expression combined with the CAFs infiltration on the prognosis of GC patients. TIMER2.0 data analysis showed that high levels of CAFs infiltration were associated with poorer prognosis in patients with gastric cancer (Figure 7A). According to the expression of NFYB, GC patients were divided into two groups: the low NFYB expression group and the high NFYB expression group. And we found no significant correlation between the infiltration degree of CAFs and the prognosis of GC patients in the low NFYB expression group, while in the high NFYB expression group, the prognosis of GC patients with high infiltration of CAFs was worse. Taken together, high expression of NFYB and high infiltration of CAFs predicted a worse prognosis for GC patients.

## DISCUSSION

GC is one of the most common malignant tumors, and its mortality and morbidity are at the forefront of malignant tumors [17]. It is of great clinical significance to explore the new molecular mechanism of GC and to find new therapeutic targets for GC. NFYB is a subunit of nuclear transcription factor Y, Also known as HAP3, CBF-A, CBF-B, and NF-YB. Its location is 12q23.3. NFYB was highly expressed in multiple tumors and promotes tumor invasion, metastasis, and drug resistance [14–16, 18]. However, the role of NFYB in gastric cancer has not been reported, so studying the effect of NFYB on GC may further clarify the possible mechanism of the occurrence and development of GC. In this study, by mining TCGA and GEO public databases, we found that NFYB is highly expressed in GC tissues and is associated with a poor prognosis of GC. There was no significant difference in the expression of NFYB in T1 gastric cancer compared with normal tissues. However, with the increase in the T stage, the expression of NFYB also increased significantly, suggesting that NFYB may be associated with the invasion and metastasis of GC. The expression of NFYB in 21-40 year-old gastric cancer patients was significantly higher than that in older gastric cancer patients, suggesting that NFYB may play a role in the occurrence and development of early-onset gastric cancer. Meanwhile, the analysis showed that the expression of NFYB was higher in poorly differentiated GC tissues and higher in diffuse gastric cancer than in intestinal gastric cancer. These results suggest that high NFYB expression may be associated with the poor prognosis of GC patients. It has been reported that CAFs are related to the occurrence, development, and prognosis of gastric cancer, but the specific mechanism of the difference in the infiltration degree of CAFs in gastric cancer patients remains unclear. Our analysis showed that the expression of NFYB was positively correlated with the degree of CAFs infiltration in gastric cancer patients. The expression of NFYB was correlated with the expression of various markers of CAFs. According to the expression of NFYB, GC patients were divided into two groups: the low NFYB expression group and the high NFYB expression group. And we found no significant correlation between the infiltration degree of CAFs and the prognosis of GC patients in the low NFYB expression group, while in the high NFYB expression group, the prognosis of GC patients with high infiltration of CAFs was worse. These results indicated that the overexpression of NFYB was closely related to the function of CAFs. Altogether, our study provides insights into understanding the potential role of NFYB in CAFs and its use as potential anti-cancer targets.

We used the TIMER database to analyze TCGA’s pan-cancer RNA-SEQ expression data and explore the expression of NFYB in various tumors. The results showed that NFYB expression was significantly higher in Cholangiocarcinoma (CHOL), colon cancer, esophageal cancer, head and neck cancer, hepatocellular carcinoma, and gastric cancer than in adjacent normal tissues. It was significantly lower expressed in breast cancer, glioma, kidney cancer, pheochromocytoma and paraganglioma, prostate cancer, skin melanoma, and endometrial cancer than in normal adjacent tissues. This suggests that NFYB may have different functions in different types of cancer. It has been reported that NFYB is highly expressed in the head and neck squamous cell carcinoma [16], lung cancer [19], cervical cancer [20], and liver cancer [18], which is consistent with our analysis results. We then analyzed the correlation between NFYB expression and clinicopathology of gastric cancer. The results showed that the expression of NFYB was correlated with T stage, age of onset, histological grade, and laurun type of gastric cancer. The expression of NFYB was relatively higher in patients with a higher T stage, suggesting that NFYB overexpression may be related to the invasion of GC. Previous studies have shown that NF-Y drives the transcription of many cell cycle regulatory genes and plays a crucial role in proliferation regulation [13]. Uncontrolled proliferation is a hallmark of cancer cells, and proper control of cell growth is crucial to preventing cancer. NFYC has been shown to promote the growth of prostate cancer cells [21]. The expression of NFYB in 21-40 year-old gastric cancer patients was significantly higher than that in older gastric cancer patients, suggesting that NFYB may play a role in the occurrence and development of early-onset gastric cancer. The potential etiology of early-onset gastric cancer is still unknown and is a severe social burden [22]. Many patients with early-onset gastric cancer were already at an advanced stage when diagnosed and could not benefit from treatment [23]. Therefore, exploring the molecular markers of early-onset gastric cancer is of great significance and realizing the early diagnosis of early-onset GC. Our results also showed that NFYB expression was correlated with diffuse gastric cancer and poor differentiation of gastric cancer cells. These results suggest that NFYB expression may be associated with a poorer prognosis in patients with gastric cancer. Hence, we used the TCGA-STAD cohort and GEO GC dataset to analyze the affection of NFYB expression levels on the prognosis of GC patients. The results showed that high expression of NFYB was associated with poor OS and DFS but not with PFS in gastric cancer patients. These results suggest that NFYB may serve as a prognostic molecular marker for gastric cancer.

To explore NFYB interacting proteins and their possible functions, we performed protein-protein interaction network analysis using the String database. The minimum interaction score was set as 0.9, and the PPI Enrichment p-value was less than 0.05. The results showed that the NFYB protein interaction network contained 11 genes with 23 edges, and the PPI enrichment p-value was 0.0033 (Figure 4A). These genes include NFYA POLE4, NFYB, EP300, NFYC, TP53, MYC, XBP1, REPIN1, CHRAC1, and CIITA. Among them, TP53, MYC, and EP300 are widely studied oncogenic genes that affect cancer’s occurrence and development in various ways [24–26]. We performed the GO and KEGG pathways enrichment analysis of NFYB interacting proteins. The results showed these genes are closely related to gene transcription regulation and lipid metabolism. It is also closely related to the Wnt signaling pathway, TGFβ signaling pathway, and JAK-Stat signaling pathway. These results suggested that NFYB may be related to tumor immune or with CAFs infiltration.

High NFYB expression was associated with GC stromal score, and about 50% of stromal cells in the tumor microenvironment were CAFs [4]. The role of CAFs in various types of cancer has been proven. And CAFs can promote cancer progression through multiple mechanisms [27, 28]. However, depletion of fibroblasts accelerates tumor growth in pancreatic cancer, and specific subpopulations of CAFs exhibit cancer suppressive effects, indicating the molecular and functional heterogeneity of CAFs [29, 30]. Nevertheless, most current studies support CAFs as a tumor-promoting agent and consider targeting CAFs may be a promising cancer treatment strategy. At present, many markers of CAFs have been identified; common markers include α-smooth muscle actin (α-SMA) and fibroblast activation protein, FAP, fibroblast specific protein1, FSP1, platelet-derived growth factor receptor beta (PDGFR - beta), etc. [31]. Specific labeled CAFs subgroups can affect the survival and prognosis of tumor patients. For example, the increase of α-SMA labeled CAFs cells in breast cancer is significantly associated with poor prognosis, and FAP+ CAFs are abundant in invasive breast cancer tumor tissue [32, 33]. PDGFR-β -labeled CAFs subpopulations are associated with a higher risk of recurrence and a poor prognosis for ductal carcinoma *in situ* [34]. Our analysis showed that the expression of NFYB was positively correlated with the CAFs infiltration in GC. However, it has been reported that NFYB can promote T cell infiltration in the tumor microenvironment [35]. Therefore, NFYB may not promote tumor development through the immunosuppressive effect of CAFs. In pancreatic ductal adenocarcinoma (PDAC), some researchers found two subsets CAFs, which were muscle fibroblasts CAFs (myCAFs) and inflammatory CAFs (iCAFs) [36]. myCAFs are characterized by high expression of α-SMA, secretion of extracellular matrix components, and rapid proliferation around tumor cells [36]. In contrast, iCAFs are characterized by low α-SMA expression, sufficient secretion of inflammatory factors, slow proliferation, and distance from tumor cells [36]. In terms of function, myCAFs mainly mediate the production of extracellular matrix components to promote tumor metastasis and invasion, while iCAFs can secrete some cytokines such as IL-1 and IL-6 to promote tumor growth and mediate immunosuppression. Therefore, we further analyzed the correlation between NFYB and CAFs molecular markers. The expression of NFYB was positively correlated with most of the molecular markers of CAFs. The correlation with FAP, α-SMA, and Vimentin was stronger. This suggested that NFYB may promote the infiltration of myCAFs in GC and promote the metastasis and invasion of GC. In the high NFYB expression group, the prognosis of GC patients with high infiltration of CAFs was worse.

In conclusion, NFYB is differentially expressed in a variety of cancers. In GC, high NFYB expression was associated with high T stage, high histological grade, and diffuse gastric cancer. Meanwhile, NFYB may be a marker molecule of early-onset gastric cancer. High expression of NFYB is associated with poor prognosis of GC and promotes CAFs infiltration in the GC microenvironment. Therefore, NFYB may serve as a potential prognostic biomarker in patients with GC.

## MATERIALS AND METHODS

### Public data acquisition

The mRNA expression data and clinicopathological and follow-up data of the TCGA-STAD cohort were downloaded from the UCSC Xena Platform (https://xenabrowser.net/datapages/). The mRNA expression data of GSE79973 and GSE118916 were downloaded from Gene Expression Omnibus (GEO) (https://www.ncbi.nlm.nih.gov/geo/).

### TIMER2.0 database analysis

We used the Gene_DE module of the TIMER2.0 database to explore the expression of NFYB in pan-cancers in the TCGA database. We used “R” software to calculate the differential expression of NFYB and the “ggplot2” package to visualize the results. The correlation of the expression of the target molecule was analyzed by the Gene_Corr Module of the TIMER2.0 database, and the correlation between the expression of NFYB and CAFs infiltration in gastric cancer was analyzed by Gene Module. The Outcome Module was used to analyze the correlation between NFYB expression and the degree of CAFs invasion, and the prognosis of GC.

### UALCAN database analysis

UALCAN is a comprehensive, user-friendly, interactive web resource for analyzing cancer OMICS data. We used the UALCAN database to analyze NFYB expression differences at different clinicopathological stratification in the TCGA-STAD cohort to explore the correlation between NFYB expression and clinicopathology of GC.

### Protein-protein interaction(PPI) analysis

Thanks to the String database’s powerful visualization and customization capabilities, we can get data results while getting a lovely graph. Therefore, we used the STRING [37] database to analyze the protein-protein interaction network of NFYB and set the minimum interaction score as 0.9 and the PPI Enrichment p-value <0.05.

### Function enrichment analysis

To explore the function of NFYB, the KEGG pathway and Gene Ontology (GO) enrichment analysis were conducted using the clusterProfiler R package (v3.0.0) [38] with the FDR cutoff of 0.05. The results were visualized using the histogram generated by the “ggplot2” [39] package.
